# Plant biosecurity threats detected using metatranscriptomic sequencing of animal gut contents

**DOI:** 10.1093/ve/veaf067

**Published:** 2025-09-05

**Authors:** Jackie E Mahar, Jonathon C O Mifsud, Kate Van Brussel, Anna E Lachenauer, Erin Harvey, Olivia M H Turnbull, Stefanie Bonat, Thomas M Newsome, Annabelle Olsson, Antje Chiu-Werner, Menna E Jones, Edward C Holmes, Solomon Maina

**Affiliations:** School of Medical Sciences, University of Sydney, Sydney, NSW 2006, Australia; CSIRO Australian Animal Health Laboratory and Health and Biosecurity, Australian Centre for Disease Preparedness, 5 Portarlington Rd, East Geelong, VIC 3219, Australia; School of Medical Sciences, University of Sydney, Sydney, NSW 2006, Australia; School of Medical Sciences, University of Sydney, Sydney, NSW 2006, Australia; School of Medical Sciences, University of Sydney, Sydney, NSW 2006, Australia; School of Medical Sciences, University of Sydney, Sydney, NSW 2006, Australia; School of Medical Sciences, University of Sydney, Sydney, NSW 2006, Australia; Centre for Molecular Oncology, Faculty of Medicine, University of New South Wales, Kensington, NSW 2052, Australia; School of Life and Environmental Sciences, University of Sydney, Sydney, NSW 2006, Australia; School of Life and Environmental Sciences, University of Sydney, Sydney, NSW 2006, Australia; Sydney School of Veterinary Science, Wildlife Health and Conservation Hospital, University of Sydney, Camden, NSW 2570, Australia; School of Natural Sciences, University of Tasmania, Hobart, TAS 7000, Australia; School of Natural Sciences, University of Tasmania, Hobart, TAS 7000, Australia; School of Medical Sciences, University of Sydney, Sydney, NSW 2006, Australia; Plant Biosecurity Research and Diagnostics, NSW Department of Primary Industries and Regional Development, Menangle, NSW 2568, Australia

**Keywords:** metatranscriptomics, *Tobamovirus*, ribgrass mosaic virus, plant viruses, biosecurity, phylogeny

## Abstract

Ribgrass mosaic virus (RMV) and related viruses of the genus *Tobamovirus* (family *Virgaviridae*) are cruciferous plant pathogens that represent a threat to global horticultural systems. In Australia, they are considered exotic biosecurity threats, and an incursion of these viruses would require rapid and strict control efforts. However, current surveillance methods for these viruses are limited. We examined whether RMV and related tobamoviruses could be detected through the analysis of mammalian gut metatranscriptomes. Accordingly, we identified five different tobamoviruses in one or more gut metatranscriptomes of the Eastern grey kangaroo, fallow deer, domestic dog, spotted-tailed quoll, feral cat, and the Tasmanian devil. One of the tobamoviruses was also detected in a tick metatranscriptome. The five tobamoviruses detected were: (i) RMV, (ii) a novel relative of RMV, (iii and iv) two highly diverse novel tobamoviruses, and (v) the plant pathogen tobacco mild green mosaic virus (TMGMV) already known to be present in Australia. Subsequent phylogenetic analysis provided information on their origin and spread through Australia. RMV was detected at multiple sites in both the Australian Capital Territory (ACT) and Tasmania, two regions separated by ~700 km of land and 200 km of water. The novel relative of RMV was detected in the ACT and New South Wales (NSW), the highly divergent novel tobamoviruses were each detected in a single state (NSW and Queensland, QLD), while TMGMV was detected in QLD. This work highlights the potential utility of metatranscriptomic sequencing of wild animal gut samples for the surveillance of biosecurity threats to native and agricultural plant species and for studying their evolution in new environments.

## Introduction

Virus disease outbreaks, epidemics, and pandemics pose a threat to agricultural and horticultural systems globally (Thresh 2006, Jones and Naidu 2019, Jones 2021). Viral diseases have been associated with impaired crop growth and vigour, leading to diminished or total loss of gross yields (Jones et al. 2021). This has been accelerated by agricultural globalization, climate change, and factors such as pesticide resistance in virus vectors that together are complicating disease management (IPPC Secretariat 2021). Tobamoviruses (genus *Tobamovirus*, family *Virgaviridae*) are among the most damaging seed-borne viruses in horticultural crops (Alishiri et al. 2013, Dombrovsky and Smith 2017, Dombrovsky et al. 2017, Zhang et al. 2022, Zamfir et al. 2023). These viruses have a single-stranded, positive-sense genome of ~6.3–6.5 kb that typically contains four open reading frames (ORFs): an RNA replicase read-through derivative (ORF1 and 2), a movement protein (MP; ORF3), and a coat protein (CP; ORF4) (Adams et al. 2017, Melcher et al. 2021). Tobamoviruses have a broad host range, with phylogenetic clustering largely reflecting host species and genome structure (Gibbs et al. 1999), and on this basis are divided into three subgroups: subgroup 1 infect solanaceous species, subgroup 2 infect Cactaceae, Cucurbitaceae, Fabaceae, Malvaceae, and Passifloraceae species, and subgroup 3 infect a range of hosts, including Cruciferae/Brassicaceae and *Plantago* (Plantaginaceae) species (Adkins et al. 2003, Chavan and Pearson 2016). Tobamoviruses have been associated with substantial economic losses linked to reduced crop yield as well as fruit damage, which triggers a significant reduction in marketability (Hanssen and Lapidot 2012, Jones and Naidu 2019). For example, tomato brown rugose fruit virus (ToBRFV) is a damaging newly emerged tobamovirus that infects tomatoes and peppers that was first identified in Jordan (Salem et al. 2016) and Israel (Luria et al. 2017), later spreading to many countries worldwide and posing a major threat to the global tomato industry (Ilyas et al. 2022, Ghorbani 2024). Similarly, cucumber green mottle mosaic virus (CGMMV) is a recently introduced tobamovirus in Australia (Tesoriero et al. 2015) that causes serious disease and damage in the *Cucurbitaceae*, such that it is one of the most economically important cucurbit viruses (Maina et al. 2016, Dombrovsky et al. 2017, Jones et al. 2022, Mulholland 2022).

Agriculture of non-native crops in Australia commenced after colonization by Europeans in 1788 and as recently as the 1960s in some regions (Jones 2009, McFarlane et al. 2013, Spiegelman and Dinesh-Kumar 2023). Such a recent timescale, as well as the isolation of this large island nation, means that many viruses and virulent virus strains that co-evolved with these plants and cause damaging crop diseases in other parts of the world are absent or have only recently arrived in Australia (Gibbs 1999, Jones 2009, Dombrovsky et al. 2017). Human activities within the global trade in plants and plant products, mostly involving unknowingly infected seed or vegetative propagules, may spread viruses from other countries to Australia (Gibbs et al. 2008, Jones 2009, IPPC Secretariat et al. 2021), including recent detections of CGMMV and pepper vein yellows virus (Tesoriero et al. 2015, Maina et al. 2016). Alternatively, viruses can spillover from indigenous wild plant populations to managed cultivated populations (Roossinck and García-Arenal 2015, Jones 2020, Xu et al. 2022). As agriculture and horticulture continues to expand in Australia, so too does the overlap of wild plant and crop habitat, increasing chances of virus spillover (Malmstrom and Alexander 2016). Animal fauna may also play a role in the transmission and spread of plant viruses. Indeed, plant viruses may colonize new environments through grazing animal teeth, ingestion of plants, and subsequent passing of the viruses through the gut (Roossinck 2013), although this is rarely studied (Roossinck 2012, Malmstrom and Alexander 2016).

Recently, we inadvertently detected ribgrass mosaic virus (RMV) in Australia in wild animal gut content while exploring animal viromes using metatranscriptomic (i.e. total RNA) sequencing (Mahar et al. 2020). RMV is considered a biosecurity risk and was previously considered to be exotic to Australia but is present in neighbouring New Zealand (Cohen et al. 2012). Its natural hosts include members of the Cruciferaceae and Plantaginaceae, and it has a broad host range compared to other tobamoviruses (Wetter 1986). RMV belongs to *Tobamovirus* subgroup 3 and is most closely related to youcai mosaic virus (YoMV), turnip vein clearing virus (TVCV), and wasabi mottle virus (WMoV) (Heinze et al. 2006), all of which are classed as exotic plant pathogens and Australian biosecurity risks (https://www.agriculture.gov.au/biosecurity-trade/pests-diseases-weeds/plant/national-priority-plant-pests-2019, accessed 21 October 2024).

Tobamoviruses are highly contagious, easily spread through mechanical transmission between plants and fomites (Smith and Dombrovsky 2019), and, in some cases, are seed-transmitted. They can also remain viable in soil for many months and are very difficult to eradicate once present (Lovelock et al. 2022). As there are no available treatments, plant destruction and soil decontamination are the only means of control (Dall et al. 2023). Consequently, early detection and prevention of incursion are key to the control of these viruses, alongside rapid deployment of eradication and management plans in the event of incursions. Current biosecurity surveillance methods in Australia rely on visual inspection, immunological assays, and molecular methods such as screening of imported seed lots (Dall et al. 2023), with efforts to include targeted plant viral genomics in development (Maina et al. 2021).

In the same manner as mammalian species act as sentinels to identify human disease risks (Hazen et al. 2024), terrestrial animals may represent an untapped resource for plant pathogen surveillance, facilitating the detection of plant pathogens before they reach farms. Herein, we explored the use of an alternative surveillance strategy based on the metatranscriptomic sequencing of animal gut content to identify plant viruses, including cryptically circulating biosecurity threats within wild or cultivated vegetation, and to reveal their evolutionary history and phylogeography. We used this approach to (i) determine the presence of biosecurity threat tobamoviruses in Australia, at the same time assessing the broad-scale utility of this method, and (ii) to reveal the phylogenetic relationships among the tobamoviruses detected and their implications for the biosecurity of the Australian horticultural industry.

## Methods

### Ethics

For deer and kangaroo carcasses, scientific licences and collection permits were obtained to relocate carcasses (SL102334) and research was approved by the University of Sydney Animal Ethics Committee, project number: 2019/1640. Dog and tick sampling was approved by the Taronga Conservation Society Australia’s Animal Ethics Committee, approval number 4c/10/21. Ethics for Tasmanian carnivores was obtained from the University of Tasmania under Animal Ethic Committee permit number A0018012. Ethics for rabbit sampling is as described in Mahar et al. (2020).

### Metatranscriptomic sequence data

Metatranscriptomic data sets previously generated from studies of Australian animal viromes were screened for the presence of tobamoviruses. These data sets were generated by sequencing total RNA extracted from whole ticks and mammalian gut content in the form of faecal samples, caecal content samples, or anal/rectal swabs (Table 1). Total RNA was extracted using the Maxwell 16 LEV simply RNA tissue kit in combination with a Maxwell nucleic acid extraction robot (Promega, WI, USA) for rabbit caecal content; the Qiagen RNeasy Plus Universal Mini Kit (Hilden, Germany) for deer and kangaroo carcass swabs; and the Qiagen RNeasy Plus Mini Kit (sometimes in conjunction with Qiagen QiaShredder columns) for dog swabs, ticks, and marsupial carnivore faecal samples. The rabbit RNA libraries were prepared using the TruSeq Total RNA library preparation protocol (Illumina, CA, USA), with ribosomal RNA (rRNA) depletion using Illumina Ribo-Zero gold epidemiology rRNA removal kit. The Illumina Stranded Total RNA Prep with Ribo-Zero Plus kit was used for all the other libraries. Paired-end sequencing was conducted on an Illumina HiSeq 2500 (200 cycles) for the rabbit libraries and an Illumina NovaSeq 6 000 S4 lane (300 cycles) for all the other libraries) (Illumina, CA, USA). All library preparation and sequencing were conducted at the Australian Genomic Research Facility (AGRF). The raw reads obtained were trimmed using Trimmomatic v0.38 (Bolger et al. 2014) and *de novo* assembled using MEGAHIT v1.2.9 (Li et al. 2015) or Trinity v2.5.1 (Grabherr et al. 2011). Further details on data generation for data sets collected from rabbits can be found in Mahar et al. (2020).

**Table 1:** Metadata of metatranscriptome libraries containing tobamoviruses

**Collection location**	**Library name**	**Animal species (common name)**	**Sample type**	**No. samples in pool**	**Collection date**	**Tobamovirus(es) detected**
Crackenback/Moonbah, NSW	12-Kang2B	*Macropus giganteus* *^b^* (Eastern grey kangaroo)	Mouth and anal swab	4	Feb 2021	SG3-like tobamovirus
Crackenback/Moonbah, NSW	13-Deer2A	*Dama dama* *^b^* (Fallow deer)	Mouth and anal swab	4	Dec 2020 and Jan 2021	SG3-like tobamovirus
Crackenback/Moonbah, NSW	15-Deer2B	*Dama dama* *^b^* (Fallow deer)	Mouth and anal swab	4	Jan 2021	Bambi tobamovirus
Crackenback/Moonbah, NSW	16-Deer2B	*Dama dama* *^b^* (Fallow deer)	Mouth and anal swab	5	Feb and Mar 2021	SG3-like tobamovirus, Bambi tobamovirus
Hope Vale, QLD	HPV3-R	*Canis lupus familiaris* (Domestic dog)	Rectal swab	2	Dec 2021	Bluey tobamovirus
Hope Vale, QLD	HPV4-R	*Canis lupus familiaris* (Domestic dog)	Rectal swab	3	Dec 2021	Bluey tobamovirus
Hope Vale, QLD	HPV6-R	*Canis lupus familiaris* (Domestic dog)	Rectal swab	1	Dec 2021	Bluey tobamovirus, TMGMV
Hope Vale, QLD	HPV15-2R	*Canis lupus familiaris* (Domestic dog)	Rectal swab	2	Feb 2022	Bluey tobamovirus
Hope Vale, QLD	HPV16-2R	*Canis lupus familiaris* (Domestic dog)	Rectal swab	3	Feb 2022	Bluey tobamovirus
Hope Vale, QLD	HPV35-2E	*Rhipicephalus sanguineus* (tick), attached to domestic dog	Whole animal	1	Feb 2022	Bluey tobamovirus
Ben Lomond, TAS	P04	*Dasyurus maculatus* (Spotted-tailed_quoll)	Faecal	2	Apr–Aug 2021	RMV
Northern Slopes, TAS	P19	*Felis catus* (feral cat)	Faecal	1	Apr–Aug 2021	RMV
Northern Slopes, TAS	P21	*Dasyurus maculatus* (Spotted-tailed_quoll)	Faecal	1	Apr–Aug 2021	RMV
Northern Slopes, TAS	P22	*Sarcophilus harrisii* (Tasmanian devil)	Faecal	5	Apr–Aug 2021	RMV
Crace, ACT	GUN-CC^a^	*Oryctolagus cuniculus* ^b^ (European rabbit)	Caecal content	20	Dec 2016–Jan 2017	RMV, SG3-like tobamovirus
Booth, ACT	Gudg-CC^a^	*Oryctolagus cuniculus* ^b^ (European rabbit)	Caecal content	18	Feb 2017	RMV

a Published in Mahar et al. (2020).

b Samples taken from carcasses, not live animals.

### Tobamovirus detection

The assembled contigs were annotated using DIAMOND BLASTx (Buchfink et al. 2021), and blastn (Altschul et al. 1990) against the National Center for Biotechnology Information's (NCBI) nonredundant protein and nucleotide databases, respectively. Contigs with top hits (i.e. lowest e-value) to tobamovirus were selected for further analysis. ORFs were identified using the Find ORFs tool within Geneious Prime® 2022.2.2 using the standard genetic code and UAG read-through and adjusted based on ORF positions of related published sequences. ORF gene assignments were confirmed by identification of conserved domains using RSP-TBLASTN v2.6.0 (Altschul et al. 1997) and the NCBI Conserved Domain Database (CDD).

### Phylogenetic analysis and classification of tobamoviruses

Contigs that had a top blast hit to a tobamovirus were confirmed through sequence alignment and phylogenetic analysis with other published tobamovirus sequences. Accordingly, the tobamovirus NCBI nucleotide RefSeqs, the top blast hits to the tobamoviruses detected here, as well as all published nucleotide sequences of RMV, TVCV, WMoV, YoMV, and TMGMV, were downloaded from the NCBI nucleotide database (https://www.ncbi.nlm.nih.gov/; accessed March 2023) and aligned with the tobamovirus nucleotide sequences detected in this study using MAFFT v7.490 (Katoh et al. 2013). Ambiguously aligned regions were removed using trimAl v1.4.1 (Capella-Gutiérrez et al. 2009). Nucleotide substitution model selection was performed (Kalyaanamoorthy et al. 2017), and maximum likelihood phylogenies were then inferred using IQ-TREE v2.1.3 (Nguyen et al. 2015). Branch supports were estimated using bootstrapping (1000 replicates). Phylogenetic trees were inferred for both the complete genome (*n* = 131, 8341 nt), as well as the CP gene, the most commonly sequenced gene among tobamoviruses (*n* = 167, 528 nt). A distance matrix for subgroup 3 tobamoviruses was inferred by aligning all available subgroup 3 nucleotide sequences from NCBI with the new sequences from this study, using MAFFT. Identical sequences were removed, and genetic distances were calculated (as percentage nucleotide identity) and visualized in Geneious Prime® 2022.2.2.

### Recombination detection

All the tobamoviruses analysed here were screened for the presence of recombination using the Recombination Detection Program v4.96 (RDP4) (Martin et al. 2021), using the default parameters. A full genome alignment including the new tobamoviruses detected here, NCBI RefSeqs for subgroup 1 and 3 viruses, and the top blast hits for each tobamovirus contig was used as input (*n* = 75 sequences). The highest acceptable *P*-value was set to .05 with Bonferroni correction, and recombination events were only considered if detected by at least three different methods.

### Meta-transcriptomic identification of potential plant host species

CCMetagen (Marcelino et al. 2020) was used to classify the nonviral transcripts in the libraries where tobamoviruses were detected, using the default method of abundance calculation (i.e. by applying an additional correction for template length). Classification was based on screening against the curated indexed database compiled by the CCMetagen creators, which contains the NCBI nucleotide collection, with the exception of most artificial and environmental sequences that lack taxa IDs.

### PCR confirmation

The presence of the novel or exotic tobamoviruses detected within each metatranscriptomic library was confirmed by amplifying a small fragment of the relevant virus using the specific primers listed in Table S1 (RMV, 565 bp; SG3-like tobamovirus, 571 bp; bluey tobamovirus, 226 bp; bambi tobamovirus, 540 bp). For RMV, the SuperScript™ One-Step RT-PCR System with Platinum™ Taq DNA polymerase (Invitrogen, MA, USA) was used according to the manufacturer’s instructions. RMV-isolate PV-051-infected lyophilized plant material imported from DSMZ Germany was used as a positive control. Optimum cycling conditions were as follows: 50°C for 30 min for reverse transcription, 95°C for 15 min followed by 35 cycles at 95°C for 30 s, 55°C for 40 s, and 72°C for 45 s, with a final extension at 72°C for 10 min. For all other viruses, the RT-PCRs were conducted in a different lab using the SuperScript™ IV One-Step RT-PCR system (Invitrogen) according to the manufacturer’s protocol. The cycling conditions were as follows: 50°C for 10 min and 98°C for 2 min, followed by 35 cycles of 98°C for 10 s, 55°C for 10 s, and 72°C for 30 s, with a final extension of 72°C for 5 min. The amplified specific PCR products generated were confirmed by gel electrophoresis followed by SYBR safe staining. At least one set of PCR products for each virus was purified and confirmed by Sanger sequencing at the AGRF.

### Amplification and Sanger sequencing confirmation of SG3-like tobamovirus virus genome

To confirm the consensus genome sequence of SG3-like tobamovirus, RT-PCR and Sanger sequencing was performed on sample 12.9 from library 16-Deer2B. The tobamovirus sequence was amplified in four segments (~1040–2100 bp) using the SuperScript™ IV One-Step RT-PCR system (Invitrogen) following the manufacturer’s protocol and using four primer sets (Table S1). The cycling conditions were as follows: 50°C for 10 min and 98°C for 2 min, 35 cycles of 98 °C for 10 s, 55°C for 10 s and 72°C for 1 min, and a final extension of 72°C for 5 min. DNA amplicons were purified using the GenElute PCR clean-up kit (Sigma-Aldrich, MO, USA) and sent for single-direction Sanger sequencing at the AGRF using the amplification primers plus additional sequencing primers (Table S1). The resulting chromatograms were trimmed and mapped to the original metatranscriptomic-assembled genome sequence using Geneious Prime version 2022.1.1, and a consensus was generated.

## Results

### Detection and classification of tobamoviruses in animal gut metatranscriptomes

**Figure 1:**
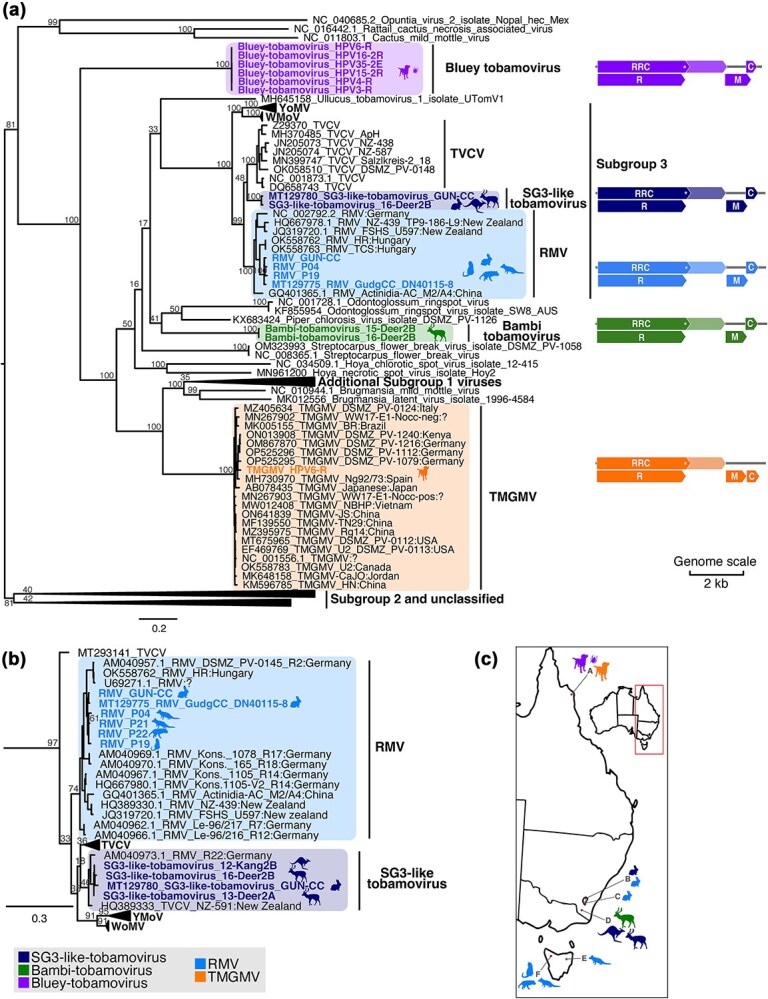
Diversity and geographic distribution of tobamoviruses found in animal metatranscriptomes in Australia. (a) ML phylogenetic tree of the genome sequences of the tobamoviruses detected in Australian animal metatranscriptomes together with published tobamovirus genomes. (b) ML phylogenetic tree of the CP gene sequences of tobamoviruses detected in Australian animal metatranscriptomes together with published CP sequences, showing only the subgroup 3 clade (the full CP phylogeny is provided in Supplementary Fig. S1). In the ML trees, sequences obtained from animal metatranscriptomes in Australia are indicated in bold and coloured by virus, and the clades that they cluster in are highlighted in the same colour. Animal silhouettes beside the clades indicate the animal metatranscriptomes from which these viruses were obtained and are coloured by virus. The GenBank accession number for published sequences is indicated at the start of the taxon name. Numbers at the nodes indicate the percentage support from 1000 bootstrap replicates, and the trees are midpoint rooted. The location (country) of collection is indicated in the taxa name in relevant clades. Genome schematics for viruses detected in this study are shown to the right of the relevant viral group, with coloured arrow-tipped boxes representing forward open reading frames for the following gene products: RNA replicase read-through component (RRC), RNA replicase (R), movement protein (M), and coat protein (C). The read-through stop codon in the RRC is indicated by an asterisk and a break in the box, while the RNA-dependent RNA polymerase region translated *via* the read-through mechanism is indicated by lighter shading. (c) Map of eastern Australia indicating where tobamoviruses were detected. Animal silhouettes indicate the animal metatranscriptomes where these viruses were obtained and are coloured by virus. Locations on the map are indicated by a letter and are as follows: A = Hope Vale, QLD; B = Crace, ACT; C = Booth, ACT; D=Crackenback/Moonbah, NSW; E = Ben Lomond, TAS; F = Northern Slopes, TAS.

Viral contigs related to tobamoviruses were detected by Blast analyses in the gut metatranscriptomes of Australian wild animals collected from four states/territories in eastern Australia: NSW, TAS, ACT, and QLD. Phylogenetic classification based on complete genome sequences (Fig. 1a) revealed the presence of five different tobamoviruses, with the top blast result for the longest contig for each of these five viruses suggesting that they be provisionally characterized as: (i) Tobacco mild green mosaic virus (TMGMV), (ii) two subgroup 3 viruses (top hits to RMV and TVCV), and (iii) two distinct novel tobamoviruses [i.e. with only 70.72% and 73.41% identity to Piper chlorosis virus (PChV) and Streptocarpus flower break virus (SFBV), respectively] (Table 2). Although tobamovirus contigs varied in length, the complete coding region of each of the five tobamoviruses detected here was assembled from at least one sequencing library.

**Table 2:** Top Blastn matches for the longest contig of each of the five tobamoviruses detected in Australian animal metatranscriptomes and their final classification

**Top Blastn hit**				
**Virus**	**Accession**	**Identity**	**Query coverage**	**Final classification (species)**
**TMGMV**	OR082766.1	98.09%	100%	TMGMV (*Tobamovirus mititessellati*)
**RMV**	OK558762.1	94.96%	100%	RMV (*Tobamovirus plantagonis)*
**TVCV**	MH370485.1	88.33%	100%	SG3-like tobamovirus (novel species)
**PChV**	ON924221.1	70.72%	53%	Bambi tobamovirus (novel species)
**SFBV**	OM323993.1	73.41%	13%	Bluey tobamovirus (novel species)

Following the blast analysis, phylogenetic analysis and level of sequence similarity was used to classify each virus more accurately. In the phylogeny, the viral sequences with top blast hits to RMV and TMGMV clustered with RMV (species *Tobamovirus plantagonis*) and TMGMV (species *T. mititessellati*) sequences, respectively (Fig. 1a), confirming the blast result. Indeed, the RMV and TMGMV detected here shared >90% sequence identity across the genome with published sequences of these viruses.

The second subgroup 3 virus that had top blast hits to TVCV formed a distinct clade that shared common ancestry with TVCV and RMV, suggesting that it may be a novel virus (Fig. 1a). In lieu of formal classification, this was tentatively named SG3-like tobamovirus. Notably, classification of the subgroup 3 viruses was hindered by the misclassification of many published virus sequences (particularly CP sequences). However, phylogenetic analysis of the complete genomes demonstrated distinct well-supported clades within the subgroup 3 tobamoviruses that corresponded to TVCV, RMV, YoMV, WMoV, and the novel SG3-like tobamovirus (Fig. 1a). The same clades were observed in phylogenetic analysis of the CP gene, although not always well supported (Fig. 1b). These phylogenies highlighted that some sequences previously published as RMV or TVCV were also likely novel SG3-like tobamoviruses, including the complete genome of a virus detected in rabbits in Australia (accession MT129780) and CP sequences from New Zealand (HQ389333) and Germany (AM040973) (Fig. 1b). Notably, as the SG3-like tobamovirus genomes did not share >90% sequence identity with any other known tobamovirus species (Table 3), these viruses would be classified as a new viral species according to ICTV demarcation criteria (https://ictv.global/report/chapter/virgaviridae/virgaviridae/tobamovirus), although this will need to be confirmed with additional data. Importantly, because this virus was novel but closely related to RMV and TVCV, the genome sequence assembled from metatranscriptomic data was confirmed by Sanger sequencing. According, the entire genome sequence was confirmed with the exception of a 395 nt region within ORF1 which could not sequenced due to insufficient sample.

**Table 3:** Whole genome nucleotide identity within and between tobamovirus subgroup 3 virus species and the novel SG3-like tobamovirus

	*T. youcai* (YoMV)^**a**^	*T. wasabi* (WMoV)	*T. rapae* (TVCV)	*T. plantagonis* (RMV)	SG3-like tobamovirus
**Maximum % identity (identical sequences removed)**					
*T. youcai*	99.9	85.0	82.4	82.5	81.7
*T. wasabi*	85.0	99.7	82.7	83.3	82.6
*T. rapae*	82.4	82.7	98.4	89.0	88.0
*T. plantagonis*	82.5	83.3	89.0	99.9	88.2
SG3-like tobamovirus	81.7	82.6	88.0	88.2	98.8
**Minimum % identity**					
*T. youcai*	89.1^b^	83.1	80.6	79.5	80.5
*T. wasabi*	83.1	98.4	81.9	80.3	82.2
*T. rapae*	80.6	81.9	92.6	85.4	87.2
*T. plantagonis*	79.5	80.3	85.4	90.3	86.0
SG3-like tobamovirus	80.5	82.2	87.2	86.0	98.8
**Mean % identity**					
*T. youcai*	94.2	84.5	81.6	81.0	81.3
*T. wasabi*	84.5	99.1	82.3	81.8	82.5
*T. rapae*	81.6	82.3	94.0	87.0	87.6
*T. plantagonis*	81.0	81.8	87.0	93.6	86.7
SG3-like tobamovirus	81.3	82.5	87.6	86.7	98.8

a Viruses belonging to species listed in parentheses.

b Minimum identity within *T. youcai* (YoMV) sequences drops below 90% due to a single sequence.

Finally, the two distinct novel tobamoviruses, tentatively named Bluey Tobamovirus and Bambi Tobamovirus, did not cluster with their top blast hits (SFBV and PChV, respectively) and were divergent from each other and all previously classified tobamoviruses (Fig. 1a). According to the ICTV demarcation criteria, these viruses would likely constitute a new viral species as they have <70% identity across the genome with any previously classified virus. These two highly novel viruses did not group into a known subgroup but were most closely related to subgroup 3 viruses, although they differed in genome structure from subgroup 3 virus (or any defined subgroup). Specifically, while defining the exact start of ORFs for novel viruses is challenging, none of the potential ORF positions result in a 77 nt CP and MP ORF overlap as seen in subgroup 3 viruses. Finally, none of the viruses discovered here were determined to be recombinants.

### Distribution, diversity, and origin of the tobamoviruses detected

TMGMV was detected in domestic dog rectal swabs from one location in QLD, while RMV was found in rabbit gut in two locations in the ACT, and in marsupial carnivore faeces in two locations in Tasmania (Fig. 1c). Genetic diversity within the detected RMVs ranged from 97.4% to 99.3% nucleotide identity. SG3-like tobamovirus was detected in both the ACT in rabbit gut and in deer and kangaroo carcass swabs in NSW, sharing 98.1%–99.9% nucleotide identity. Bluey tobamovirus was detected in a single location in QLD in five libraries sequenced from domestic dog rectal swabs, as well as a library from a tick that was attached to a domestic dog. The bluey tobamoviruses discovered here shared 98.1%–100% nucleotide identity, including the tick virus that exhibited 100% identity with a virus from a dog rectal swab. Bambi tobamovirus was found in two libraries from fallow deer anal and mouth swabs, both libraries sampled from the same deer, 2–4 weeks apart at a single NSW location, and these genome sequences exhibited 99.8% nucleotide identity.

For each virus detected here, the Australian sequences formed a monophyletic group suggestive of a single-entry event (Fig. 1a). The closest exotic relatives to the Australian RMVs were sampled from Germany and Hungary (according to the CP phylogeny, which has the most available sequence data, Fig. 1b), while the closest relatives of the SG3-like tobamoviruses were from Germany and New Zealand. The Australian TMGMV clustered with viruses from Italy and Japan, based on the CP gene tree (Supplemental Fig. S1) but was most closely related to viruses from Germany in the full genome phylogeny. Accession OP525296, from Germany, shared the highest identity with the virus detected here at 96.7% nucleotide identity across the complete genome, although there are a range of viruses from various countries that share >96% identity, including those from China (MZ395975) and Kenya (ON013908) that share 96.6% and 96.5% identity, respectively. Indeed, the most divergent TMGMV (MH730970) still shares 94.5% identity with the new Australian isolate. An additional phylogenetic analysis of TMGMVs that included sequences of historical TMGMVs sampled in Australia between 1907 and 1993 did not provide sufficient resolution to definitively reveal phylogenetic relationships (i.e. low bootstrap support values at key nodes), likely because the historical sequences only comprised two amplicons of <350 nt each (Supplementary Fig. S2).

### Abundance of plant transcripts in gut metatranscriptomes

**Figure 2:**
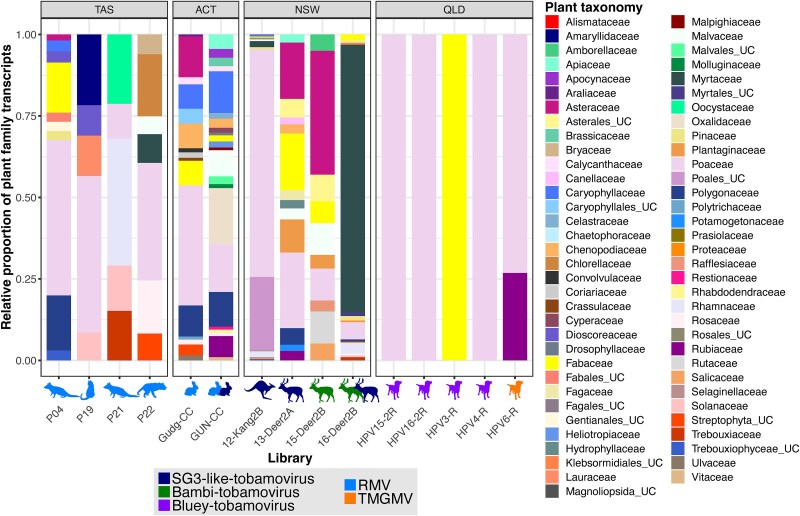
Abundance of plant transcripts in animal gut metatranscriptomes. Abundance of plant families is represented as a proportion of total plant transcripts present in each library. Animal silhouettes underneath each bar indicate the animal metatranscriptomes from which the plant transcripts were obtained and are coloured by virus. Multiple animal silhouettes represent multiple viruses in that library. Note that libraries not pictured in the plot did not contain any plant transcripts.

To assess potential hosts of the tobamoviruses detected here, we examined the metatranscriptomic data to identify transcripts from plants (Fig. 2). The Poaceae family (grasses) were detected almost universally in the dog libraries isolated in QLD. This was the only plant family detected for three of the five libraries sequenced and was only absent in one library, which was made up entirely of Fabaceae (legumes). Thus, the Poaceae and Fabaceae are strong candidates as hosts for bluey tobamovirus. Notably, grasses were present in most metatranscriptomes sampled in this study (Fig. 2), albeit in lower proportions than in the dog libraries. The libraries from TAS, ACT, and NSW contained a greater variety of plant transcripts, making it harder to identify potential hosts (Fig. 2). Both libraries containing bambi tobamovirus had transcripts from the Fabaceae, Plantaginaceae, and Poaceae but among a range of many other potential hosts. Most RMV- and SG3-like tobamovirus-containing libraries prepared from samples sourced from herbivores (with the exception of Gudg-CC and 12-Kang2B) contained Plantaginaceae and/or Brassicaceae transcripts (among a range of other plant transcripts), which are known hosts for SG3 tobamoviruses (Heinze et al. 2006, Cohen et al. 2012). However, the Plantaginaceae and Brassicaceae families were absent in the RMV-containing carnivore libraries, suggesting potential alternate hosts (Fig. 2). There were no detectable plant transcripts in the tick library.

### PCR confirmation

To verify the presence of novel and exotic viruses in each individual library, we employed RT-PCR to screen original RNA samples where available (RNA from libraries P19 and P21 was depleted, and the ACT samples were not available). Importantly, where RNA was available for testing, we confirmed the presence of all viruses detected in metatranscriptomes in one or more samples pooled in the library, including confirming the presence of the bluey tobamovirus in tick samples (Table 4). While insufficient RNA precluded the use of RT-PCR to confirm that the RMV contigs in P19 and P21 are not the result of index hopping from other libraries, the contigs from these libraries are not identical to those from other libraries sequenced on the same lane. Equally, while RMV was detected in both libraries from the ACT sequenced on the same lane, the RMV contigs in these two libraries (Gudg-CC and GUN-CC) were not identical. RT-PCR testing was not conducted for TMGMV as it was found in a single library that comprised a single sample.

**Table 4:** RT-PCR confirmation of detected viruses

**Library and RT-PCR testing details**						**No. of samples testing positive by RT-PCR per library**			
State	Library	Animal(s) sampled	No. samples in library	No. samples tested^a^	Viruses tested^b^	RMV	SG3-like tobamovirus	Bambi tobamovirus	Bluey tobamovirus
NSW	12-Kang2B	Eastern grey kangaroo	4	4	SG3-like tobamovirus	NA^c^	1	NA	NA
NSW	13-Deer2A	Fallow deer	4	4	SG3-like tobamovirus	NA	1	NA	NA
NSW	15-Deer2B	Fallow deer	4	4	Bambi tobamovirus	NA	NA	1	NA
NSW	16-Deer2B	Fallow deer	5	5	Bambi tobamovirus, SG3-like tobamovirus	NA	4	2	NA
QLD	HPV3-R	Domestic dog	2	2	Bluey tobamovirus	NA	NA	NA	2
QLD	HPV4-R	Domestic dog	3	3	Bluey tobamovirus	NA	NA	NA	3
QLD	HPV6-R	Domestic dog	1	1	Bluey tobamovirus	NA	NA	NA	1
QLD	HPV15-2R	Domestic dog	2	2	Bluey tobamovirus	NA	NA	NA	2
QLD	HPV16-2R	Domestic dog	3	3	Bluey tobamovirus	NA	NA	NA	3
QLD	HPV35-2E	Tick	1	1	Bluey tobamovirus	NA	NA	NA	1
TAS	P04	Spotted-tailed quoll	2	1	RMV	1	NA	NA	NA
TAS	P19	Feral cat	1	0	RMV	Insuff^d^	NA	NA	NA
TAS	P21	Spotted-tailed quoll	1	0	RMV	Insuff	NA	NA	NA
TAS	P22	Tasmanian devil	5	5	RMV	3	NA	NA	NA

a No. samples tested refers to the number of samples from this library tested by RT-PCR (as some samples had insufficient RNA).

b Viruses tested refers to the viruses tested by RT-PCR for this library: determined by those found in the metatranscriptome of this library as well as other libraries on the same lane (TMGMV was not included in testing as it was not a novel virus and was only found in a single library).

c NA = testing not required.

d Insuff = insufficient RNA for all samples in library (in both cases each library only had one RNA sample).

## Discussion

Tobamoviruses can have devastating impacts on agricultural and horticultural crops. Consequently, they are listed as biosecurity threats in many countries, including Australia. Australia imports seeds for the horticultural industry under stringent phytosanitary inspections, although it remains vulnerable to the introduction of seed or vector-transmitted viruses. Surveillance to monitor virulent viruses and their vectors has been critical for the early detection of exotic viruses (Maina and Jones 2023). Current surveillance methods in Australia mainly involve the use of targeted molecular approaches and immunoassays applied to survey particular field crops and samples at the border (Dall et al. 2023). However, these approaches are unlikely to detect unknown viruses and potential endemic variants and may fail to detect incursions before cultivated crops are impacted (Dombrovsky et al. 2017, Roberts et al. 2018). Combining surveillance in field crops and border samples with metatranscriptomic surveillance of wild and domestic animal gut material offers an alternative surveillance strategy for the detection of known and novel viruses, as well as exotic (or presumed exotic) virus incursions before they establish and further spread in cultivated field crops. This approach also provides a means to study their subsequent spread and evolution. Herein, the metatranscriptomic sequencing of opportunistically sampled wild and domestic animal gut material in Australia revealed five different tobamoviruses, including one classified as exotic (RMV) and three novel viruses (a tentative novel relative of subgroup 3 tobamoviruses—SG3-like tobamovirus—and two highly diverse novel tobamoviruses, bluey tobamovirus and bambi tobamovirus). This is the first study to explore the use of metatranscriptomic sequencing of vertebrate animals as a strategy to detect exotic and novel plant viruses that may pose a risk to crops.

The detection of RMV in native and feral animal gut metatranscriptomes is of importance as this virus was previously considered to be exotic in Australia (https://www.agriculture.gov.au/biosecurity-trade/pests-diseases-weeds/plant/national-priority-plant-pests-2019). Likewise, the detection of the SG3-like tobamovirus is also consequential, as its closest relatives, RMV and TVCV, are known plant pathogens (Cohen et al. 2012). In addition, this virus clusters within the larger subgroup 3 clade of viruses containing RMV, TVCV, WMoV, and YoMV, all of which are important pathogens of Brassicaceae and Plantaginaceae (Aguilar et al. 1996, Cohen et al. 2012, Kim et al. 2010, MacDonald et al. 2021, Melcher 2003). It is therefore possible that this virus could also affect crops within these plant families. Adding credence to this, the New Zealand virus that clusters with the SG3-like tobamovirus based on CP sequence (accession HQ389333, originally characterized as TVCV), was found in a *Plantago* species presenting with mild chlorosis or mottles (Cohen et al. 2012). Clearly, a broader survey of the SG3-like tobamoviruses in various hosts should be conducted, along with virus host studies.

The detection of TMGMV is not alarming as this virus is among a number of tobamoviruses that have previously been detected in Australia (Wei et al. 2012, Wylie et al. 2014, Tesoriero et al. 2015, Roberts et al. 2018, Lovelock et al. 2020). Indeed, TMGMV has been present in Australia since the late 1800s (Fraile et al. 1997). This virus seems well adapted to members of *Nicotiana* (de Andrés-Torán et al. 2023) and is particularly common in wild *Nicotiana glauca* wherever it is present around the world (Fraile et al. 1997). However, TMGMV is known to infect a wide range of hosts, including members of the Solanaceae, Umbelliferae, Gesneriaceae, Rubiaceae, and Poaceae (Wetter 1986, Zamfir et al. 2023), and transcripts of the latter two were present in the TMGMV containing library, suggesting these as potential hosts in this case.

It is difficult to speculate on the pathogenic potential or likely hosts of the two highly novel viruses detected in this study—bluey tobamovirus and bambi tobamovirus—as they were distinct from known viruses. Analysis of the eukaryotic transcripts in the libraries containing these viruses suggests grasses (Poaceae) and potentially legumes (Fabaceae) as strong candidate hosts for both novel viruses, along with Plantaginaceae for bambi tobamovirus. It is also possible that the true natural host for these viruses were not detectable due to low abundance or RNA degradation, as eukaryotic RNA transcripts would not be protected like encapsulated viral RNA. As these viruses have not been detected elsewhere, is it possible that the natural hosts of bluey tobamovirus and bambi tobamovirus are wild Australian native plants, as shown for yellow tailflower mild mottle virus (Wylie et al. 2014). As a tobamovirus originating from native Australian plants could naturally spread to exotic plant species (Xu et al. 2022), a broader understanding of the distribution and the natural hosts of these viruses could be beneficial for both native conservation and their management to deter potential spillover or spread to cultivated crops.

We detected these viruses in a variety of animal guts, including native and feral wild-living species and domestic animals, and assume that the viral hosts are plants within the animal diet. Interestingly, we also found some of these viruses in carnivores and hypothesize that the viruses were in plants that were incidentally consumed or in the gut of their ingested prey. As tobamoviruses are spread by mechanical transmission (Smith and Dombrovsky 2019), it is likely that vertebrate species may play a role in the spread of these viruses. Plant viruses are known to colonize new environments through grazing animal teeth, ingestion of plants, and subsequent passing of the viruses through the gut (Roossinck 2013). As such, these data emphasize the potential ecological importance of wild animals in the spread of tobamoviruses and other contact-transmitted viruses.

Interestingly, bluey tobamovirus was found at high abundance in a tick metatranscriptome with a near identical viral genome sequence (99.8%–100% nucleotide identity) to those found in dog faeces. While we cannot exclude an animal tropism, tobamoviruses have not been demonstrated to replicate in ticks or dogs. Further, it is not uncommon for plant viruses to be detected in (and transmitted by) invertebrates (Roossinck 2013, Roberts et al. 2018, Mahar et al. 2020), and tobamoviruses have previously been found in tick viromes in China, including Tobacco mosaic virus, YoMV, and CGMMV (Ni et al. 2023). Indeed, 80% of plant viruses rely on insect vectors for mechanical transmission (Hohn 2007), and invertebrate pollinators (such as the honeybee and bumblebee) have been shown to transmit tobamoviruses (Okada et al. 2000, Darzi et al. 2018). It is therefore possible that the bluey tobamovirus mechanically adhered to tick body parts following tick contact with infected sap, as known to occur with plant viruses transmitted by pollinator insects (Sdoodee and Teakle 1993, Okada et al. 2000, Aramburu et al. 2010, Darzi et al. 2018). As such, it is possible that ticks could play a minor role in dispersal of tobamoviruses. Finally, as the dog and tick sampling was done at an outdoor pop-up vet clinic in regional Queensland, it is also possible that the dog and tick samples were contaminated at the point of sampling.

These data demonstrate that total RNA-sequencing of animal gut material is a potentially useful additional strategy for the surveillance of many economically important plant viruses, automatically providing an insight into their origins and patterns of spread in a new environment. While current surveillance methods involve direct sampling of individual plants or seed lots, gut metatranscriptomics allows a larger-scale sampling approach whereby a pool of animal faeces, representing an entire range of ingested plants can be sequenced. Importantly, tobamoviruses were detectable regardless of the method of sampling the gut content (i.e. anal/rectal swab, faecal sample, gut content sample). In addition, where RNA extraction from plant material can be challenging, the plant material in animal gut has already been broken down, and therefore, extraction from animal material could be a simpler process requiring less complex tissue disruption methods. Conversely, viruses that are not highly stable in the environment may be less likely to be detected in metatranscriptomes (Roossinck 2013) due to degradation within the animal gut. It may also often fail to detect DNA viruses as only RNA transcripts from these viruses are expected to be sequenced and these may be degraded in the animal gut since they would not be protected by capsids. However, as RNA viruses are the most abundant plant pathogens (Pagán et al. 2010, Scholthof et al. 2011, Tatineni and Hein 2023), this approach could be a beneficial addition to current surveillance techniques. Indeed, we suggest that equivalent screens for plant pathogens present in gut metatranscriptomes be made in other jurisdictions. While this approach is unlikely to rapidly detect viral incursions as it requires at least low-level circulation of these viruses in the environment, it does represent alternative surveillance opportunity to detect viruses before they impact grower farms. Interestingly, metatranscriptomics has also been employed to detect plant viruses on honeybees in Australia (Roberts et al. 2018), including the devastating CGMMV known to have caused major losses in the horticultural industry (Mackie et al. 2023), further demonstrating the utility of metatranscriptomics of animals for plant virus surveillance.

Due to a lack of global sequence data, it is difficult to determine which viruses represent exotic introductions and the exact source of their introduction into Australia. Despite this, our phylogenetic analysis indicates that there has been at least one recent incursion event of a known virus—RMV. In addition, the fact that the Australian RMV sequences formed a monophyletic group is suggestive of a single incursion of this virus. The SG3-like tobamovirus also seems likely to be from an exotic incursion as it is closely related to New Zealand and German sequences (although with relatively weak phylogenetic support). As with RMV, the Australian sequences form a monophyletic group that is indicative of a single incursion with subsequent within-country spread. Since there is so little sequence data available, we cannot speculate on the geographic origins and distribution of this virus. Although phylogenetics serves as a critical tool for tracing the transmission of viral pathogens across national borders, its effectiveness is dependent on the availability of sufficient genomic data. Expanding genomic sequencing efforts for viral plant pathogens across all countries would greatly improve our ability to assess the distribution of understudied viruses, as well as the frequency and timing of intercountry transmission, providing valuable insights into biosecurity vulnerabilities.

Importantly, tobamoviruses were detected in animal samples from four Australian states and territories, and RMV and SG3-like tobamoviruses were each found in two states and territories, indicating spread of these viruses within Australia. RMV was detected in both the ACT and Tasmania, even though these locations are >700 km apart and separated from the mainland of Australia by Bass Strait, >200 km of water. Viruses may have spread over Bass Strait through their ability to be contact transmitted *via* fomites (Dombrovsky et al. 2017), potentially on shoes or equipment travelling by air or on car tyres on ferries, or even by migratory birds. Future phylogeographic studies are needed to provide a more detailed picture of the spread of RMV and the other tobamoviruses detected here.

In sum, we show that the metatranscriptomic screening of animal gut content could serve as a tool for the nontargeted surveillance of plant viruses. Future work should focus on characterizing the host range, geographic distribution, and impact potential of the new and known tobamoviruses detected here. This study also highlights the importance of continuous integration of innovative diagnostic and surveillance genomics tools to support viral disease management for sustainable food production and for studying the subsequent virus evolution and mechanisms of dispersal.

## Supplementary Material

Figure_S1_veaf067

Figure_S2_veaf067

Table_S1_veaf067

Mahar_Supplementary_revised_2_veaf067

## Data Availability

The consensus sequences for tobamoviruses assembled in this study have been deposited in NCBI/GenBank under the assigned accession numbers PV005927-PV005943.
