# Supplementary material for: Plant biosecurity threats detected using metatranscriptomic sequencing of animal gut contents
Source: Virus Evol. 2025 Sep 5;11(1):veaf067. doi: 10.1093/ve/veaf067 (PMC12461698; doi:10.1093/ve/veaf067)
Supplement: Figure_S1_veaf067 [file figure_s1_veaf067.pdf]

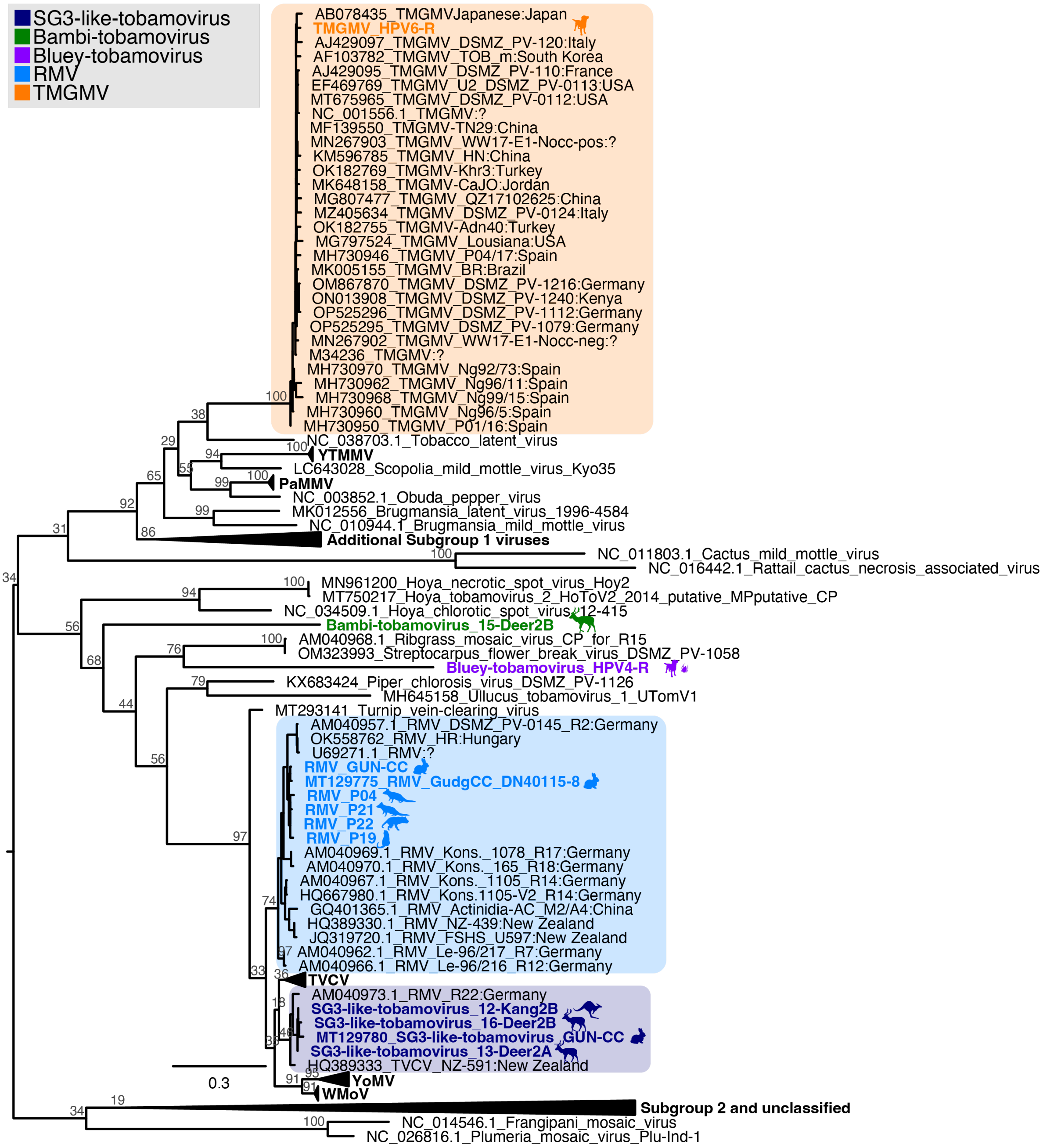

Figure S1. ML phylogenetic tree of the coat gene sequences of tobamoviruses detected in Australian animal metatranscriptomes together with coat sequences. Sequences obtained from animal metatranscriptomes in Australia are indicated in bold and coloured by virus, and the clades that they cluster within are highlighted in the same colour: purple=bluey tobamovirus; dark blue=SG3-like tobamovirus; blue=RMV; green=bambi tobamovirus; orange=TMGMV. Animal silhouettes beside the clades indicate the animal metatranscriptomes from which the viruses were obtained and are coloured by virus. For viruses with 100% identity in the coat gene only a single taxon was included, although multiple animal silhouettes may be used to indicate the range of metatranscriptomic sources. The GenBank accession number for published sequences is indicated at the start of the taxon name. Numbers at the nodes indicate the percentage support from 1,000 bootstrap replicates and the trees are midpoint rooted. The location (country) of collection is indicated in the taxa name in relevant clades.
