# Supplementary material for: Plant biosecurity threats detected using metatranscriptomic sequencing of animal gut contents
Source: Virus Evol. 2025 Sep 5;11(1):veaf067. doi: 10.1093/ve/veaf067 (PMC12461698; doi:10.1093/ve/veaf067)
Supplement: Figure_S2_veaf067 [file figure_s2_veaf067.pdf]

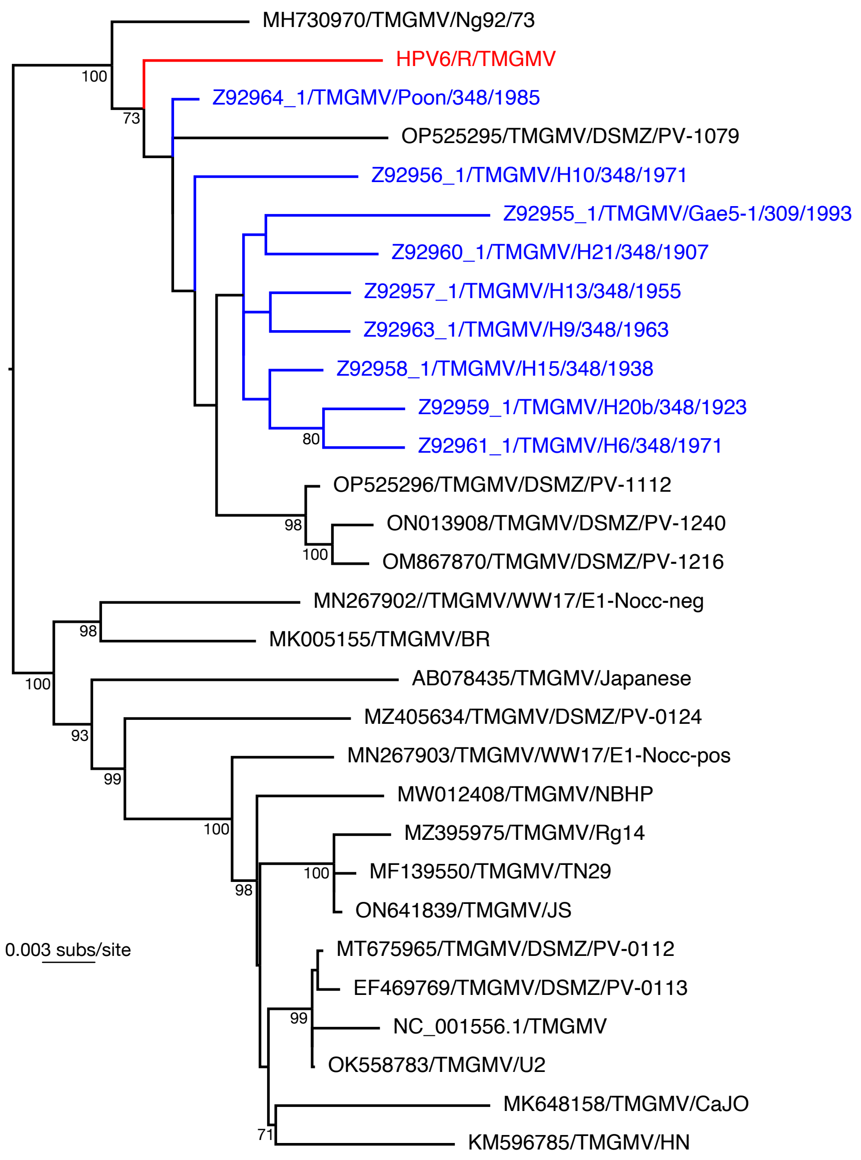

Figure S2. ML phylogenetic tree of the TMGMV genome identified here alongside global and historical Australian sequences (n=30, sequence alignment of 6575 nt). Tip labels include GenBank accession numbers and are coloured according to source: red = TMGMV detected in this study from animal metatranscriptomes; blue = previously published TMGMV from Australia (Fraile et al. 1997, including sample dates in years as tge final number in the sequence name); black = previously published TMGMV sequences from other countries. Node numbers represent bootstrap support if >70% (from 1,000 replicates). The sequences from Fraile et al. 1997 et al. span nucleotide regions 962-1309 and 3527-3833.
