# Supplementary material for: Plant biosecurity threats detected using metatranscriptomic sequencing of animal gut contents
Source: Virus Evol. 2025 Sep 5;11(1):veaf067. doi: 10.1093/ve/veaf067 (PMC12461698; doi:10.1093/ve/veaf067)
Supplement: Table_S1_veaf067 [file table_s1_veaf067.pdf]

**Table S1. Primer sets used for RT-PCR confirmation of viruses and amplification and Sanger sequencing of SG3-like tobamovirus**

| Primer name | Primer sequence 5'-3' | Direction | Target | Purpose | Amplicon size |
|---|---|---|---|---|---|
| **Full genome amplification and Sanger sequencing of SG3-like tobamovirus** | | | | | |
| FP1b | ACAAACAACAACAACATGGC | Forward | SG3-like tobamovirus | RT-PCR/Sanger | 2,003 bp |
| RP1 | AACTTGTCGAAACCACCACCT | Reverse | | RT-PCR | |
| FP2 | CGACGGAGGAGGAGATTTCAG | Forward | SG3-like tobamovirus | RT-PCR/Sanger | 1,484 bp |
| RP2 | GACCCCTGCTTCGACTTTGT | Reverse | | RT-PCR | |
| FP3 | GACGTACGAGAAGACTGCGA | Forward | SG3-like tobamovirus | RT-PCR/Sanger | 2,106 bp |
| RP3 | CGGTTTCCTCTTAGCGTCCA | Reverse | | RT-PCR | |
| FP4 | ACTTCTGCGGTCGTTACGTT | Forward | SG3-like tobamovirus | RT-PCR/Sanger | 1,723 bp |
| RP4 | CGGGGTTAGGGAGGATTCGA | Reverse | | RT-PCR | |
| FT1 | AGCGCTACTCAGAAAGAACGT | Forward | SG3-like tobamovirus | Sanger | N/A |
| FT2 | AAGTTGGGTCATGTGCAGGA | Forward | SG3-like tobamovirus | Sanger | N/A |
| FT3 | ACATGGTTTAGGGTGGCTGT | Forward | SG3-like tobamovirus | Sanger | N/A |
| FT4 | ACAGTTGGTCAGTTAGCGGA | Forward | SG3-like tobamovirus | Sanger | N/A |
| FT5 | TGGACGCTAAGAGGAAACCG | Forward | SG3-like tobamovirus | Sanger | N/A |
| FT6 | AGGGTCCCGGGTGTATGTTA | Forward | SG3-like tobamovirus | Sanger | N/A |
| **Detection RT-PCR** | | | | | |
| RMV_410_F | GCGATAAGTGATCCGGACGT | Forward | RMV | RT-PCR | 565 bp |
| RMV_974_R | ACTTCTAATGGCGACGGTCG | Reverse | | | |
| F_Bamb | AGATTGGAGAAGGGTTGCGG | Forward | Bambi tobamovirus | RT-PCR | 540 bp |
| R_Bamb | TCAGCAGGACATCGCAAAGT | Reverse | | | |
| F_Blue | CTACGTGTAGCCGTCTCGAC | Forward | Bluey tobamovirus | RT-PCR | 226 bp |
| R_Blue | TGCGAATCATTTCAGCAGCG | Reverse | | | |
| F_Novel-SG3 | AATTGGAGGAAGGGATGTGGTT | Forward | SG3-like tobamovirus | RT-PCR | 571 bp |
| R_Novel-SG3 | AAGCCTCAAACTCTGCCYTG | Reverse | | | |
