# Supplementary material for: Plant biosecurity threats detected using metatranscriptomic sequencing of animal gut contents
Source: Virus Evol. 2025 Sep 5;11(1):veaf067. doi: 10.1093/ve/veaf067 (PMC12461698; doi:10.1093/ve/veaf067)
Supplement: Mahar_Supplementary_revised_2_veaf067 [file mahar_supplementary_revised_2_veaf067.pdf]

Table S1. Primer sets used for RT-PCR confirmation of viruses and amplification and Sanger sequencing of SG3-like tobamovirus

Primer name	Primer sequence 5'-3'	Direction	Target	Purpose	Amplicon size
**Full genome amplification and Sanger sequencing of SG3-like tobamovirus**					
FP1b	ACAAACAACAACAACATGGC	Forward	SG3-like tobamovirus	RT-PCR/Sanger	2,003 bp
RP1	AACTTGTCGAAACCACCACCT	Reverse		RT-PCR	
FP2	CGACGGAGGAGGAGATTTCAG	Forward	SG3-like tobamovirus	RT-PCR/Sanger	1,484 bp
RP2	GACCCCTGCTTCGACTTTGT	Reverse		RT-PCR	
FP3	GACGTACGAGAAGACTGCGA	Forward	SG3-like tobamovirus	RT-PCR/Sanger	2,106 bp
RP3	CGGTTTCCTCTTAGCGTCCA	Reverse		RT-PCR	
FP4	ACTTCTGCGGTCGTTACGTT	Forward	SG3-like tobamovirus	RT-PCR/Sanger	1,723 bp
RP4	CGGGGTTAGGGAGGATTCGA	Reverse		RT-PCR	
FT1	AGCGCTACTCAGAAAGAACGT	Forward	SG3-like tobamovirus	Sanger	N/A
FT2	AAGTTGGGTCATGTGCAGGA	Forward	SG3-like tobamovirus	Sanger	N/A
FT3	ACATGGTTTAGGGTGGCTGT	Forward	SG3-like tobamovirus	Sanger	N/A
FT4	ACAGTTGGTCAGTTAGCGGA	Forward	SG3-like tobamovirus	Sanger	N/A
FT5	TGGACGCTAAGAGGAAACCG	Forward	SG3-like tobamovirus	Sanger	N/A
FT6	AGGGTCCCGGGTGTATGTTA	Forward	SG3-like tobamovirus	Sanger	N/A
**Detection RT-PCR**					
RMV_410_F	GCGATAAGTGATCCGGACGT	Forward	RMV	RT-PCR	565 bp
RMV_974_R	ACTTCTAATGGCGACGGTCG	Reverse			
F_Bamb	AGATTGGAGAAGGGTTGCGG	Forward	Bambi tobamovirus	RT-PCR	540 bp
R_Bamb	TCAGCAGGACATCGCAAAGT	Reverse			
F_Blue	CTACGTGTAGCCGTCTCGAC	Forward	Bluey tobamovirus	RT-PCR	226 bp
R_Blue	TGCGAATCATTTCAGCAGCG	Reverse			
F_Novel-SG3	AATTGGAGGAAGGGATGTGGTT	Forward	SG3-like tobamovirus	RT-PCR	571 bp
R_Novel-SG3	AAGCCTCAAACTCTGCCYTG	Reverse			

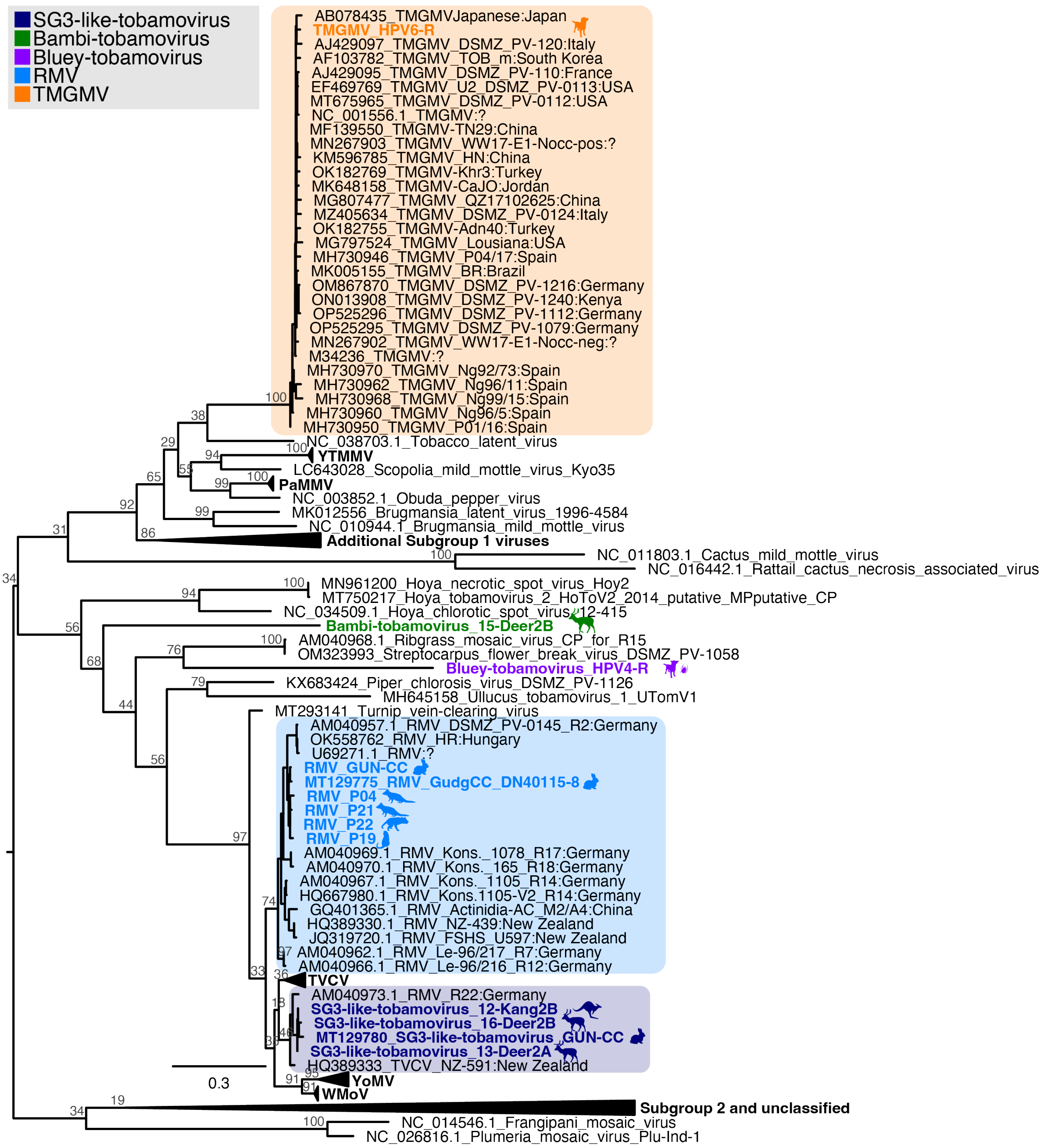

Figure S1. ML phylogenetic tree of the coat gene sequences of tobamoviruses detected in Australian animal metatranscriptomes together with coat sequences. Sequences obtained from animal metatranscriptomes in Australia are indicated in bold and coloured by virus, and the clades that they cluster within are highlighted in the same colour: purple=bluey tobamovirus; dark blue=SG3-like tobamovirus; blue=RMV; green=bambi tobamovirus; orange=TMGMV. Animal silhouettes beside the clades indicate the animal metatranscriptomes from which the viruses were obtained and are coloured by virus. For viruses with 100% identity in the coat gene only a single taxon was included, although multiple animal silhouettes may be used to indicate the range of metatranscriptomic sources. The GenBank accession number for published sequences is indicated at the start of the taxon name. Numbers at the nodes indicate the percentage support from 1,000 bootstrap replicates and the trees are midpoint rooted. The location (country) of collection is indicated in the taxa name in relevant clades.

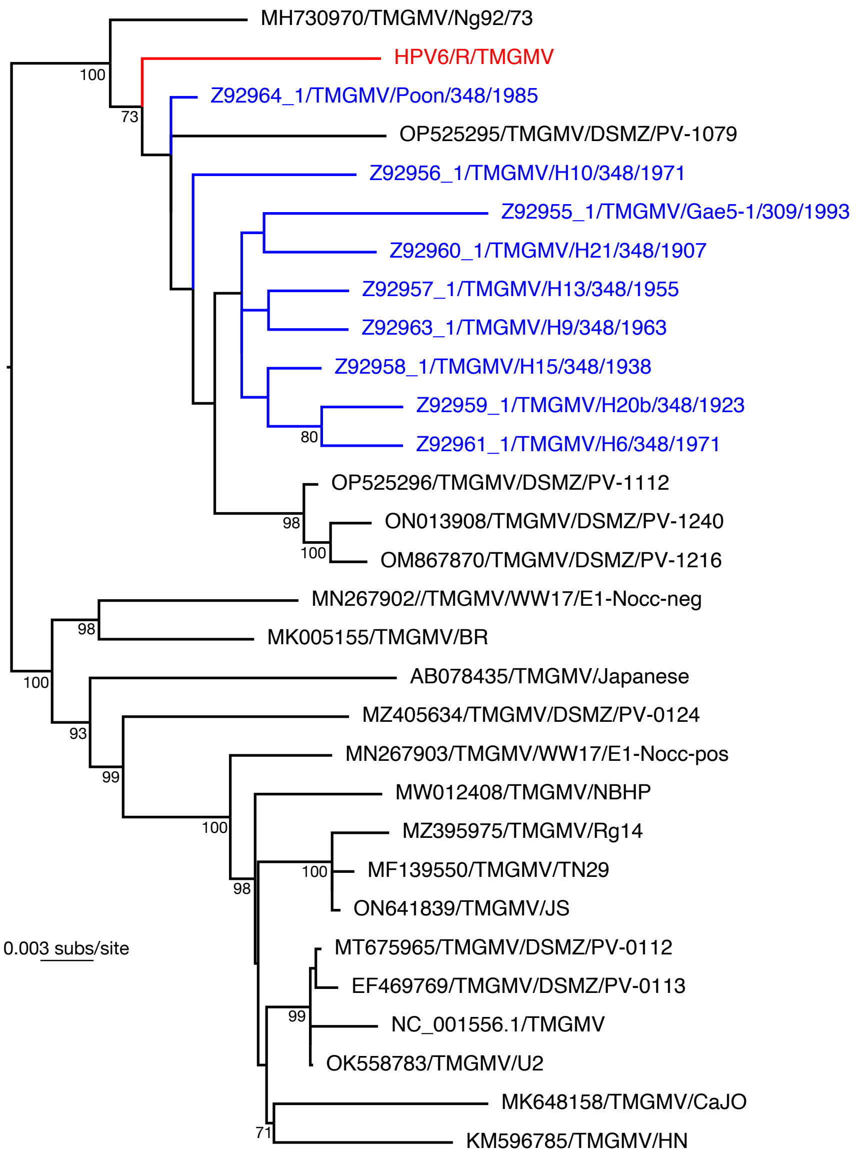

Figure S2. ML phylogenetic tree of the TMGMV genome identified here alongside global and historical Australian sequences (n=30, sequence alignment of 6575 nt). Tip labels include GenBank accession numbers and are coloured according to source: red = TMGMV detected in this study from animal metatranscriptomes; blue = previously published TMGMV from Australia (Fraile et al. 1997, including sample dates in years as tge final number in the sequence name); black = previously published TMGMV sequences from other countries. Node numbers represent bootstrap support if >70% (from 1,000 replicates). The sequences from Fraile et al. 1997 et al. span nucleotide regions 962-1309 and 3527-3833.
